# Mesonephric adenocarcinoma of the cervix: a case report with a three-year follow-up, lung metastases, and next-generation sequencing analysis

**DOI:** 10.1186/s13000-019-0847-8

**Published:** 2019-07-03

**Authors:** Nelson Montalvo, Ligia Redrobán, David Galarza

**Affiliations:** 1 Facultad de Ciencias Médicas de la Salud y la Vida, Escuela de Medicina, Universidad Internacional del Ecuador. Servicio de Patología, Hospital Metropolitano, Av. Mariana de Jesús s/n y Nicolás Arteta, Quito, Ecuador; 2 Servicio de Patología Hospital Metropolitano, Quito, Ecuador; 3 Facultad de Ciencias Médicas de la Salud y la Vida, Escuela de Medicina, Docencia y Departamento de Investigación, Universidad Internacional del Ecuador, Quito, Ecuador

**Keywords:** Cervix, Mesonephric carcinoma, Mesonephric adenocarcinoma, CTNNB1, KRAS, 1q gain

## Abstract

**Background:**

Mesonephric adenocarcinoma (MNAC) is a rare tumor of the female genital tract, which originates from mesonephric duct remnants. Its diagnosis is pathologically challenging, because MNAC may exhibit a mixture of morphological patterns that complicates the differential diagnosis.

**Case presentation:**

The patient in this case was a 48-year-old woman with a polypoid mass protruding into the endocervical canal. The patient underwent a total hysterectomy outside the institution. During biopsy, the mass showed a cerebroid aspect. Histological study revealed a tumor with a predominantly tubular and ductal growth pattern. The immunoprofile showed negative staining for calretinin, carcinoembryonic antigen (CEAm), estrogen receptors (ER), and progesterone receptors (PR), and positive staining for CD10, p16, and PAX2. The Ki-67 score was 46%. Using a next-generation sequencing assay, we documented genomic alterations in *KRAS* and *CTNNB1*, low tumor mutation burden (TMB), and an absence of microsatellite instability. In addition, gain of the long arm of chromosome 1 (1q) was also documented using chomogenic in situ hybridization (CISH). Three years later, the patient presented pulmonary nodules in the lingula and left basal lobe that were resected by thoracotomy. The histopathologic study of the pulmonary nodules confirmed the presence of metastases.

**Conclusion:**

Carcinomas of mesonephric origin are among the rarest subtypes of cervical tumors. We report the first case of mesonephric adenocarcinoma of the cervix with lung metastases showing a *CTNNB1* gene mutation.

## Introduction

Mesonephric adenocarcinoma (MNAC) is a rare tumor of the female genital tract mainly occurring in the lateral wall of the cervix and originating from mesonephric duct remnants [1, 2]. Less than one hundred cases have been reported in the literature, including tumors arising from the cervix and uterine corpus (See Table 1). Its diagnosis is pathologically challenging, because MNAC may exhibit a mixture of morphological patterns that invite misinterpretation as a benign lesion like mesonephric hyperplasia, or as a different malignant lesion of the cervix, such as endometrial endometrioid adenocarcinoma or usual-type endocervical adenocarcinoma [3, 4]. We present the first case of mesoneprhic adenocarcinoma of the cervix with a *CTNNB1* gene mutation demonstrated by NGS analysis.

**Table 1:** Summary of cases of mesonephric carcinoma of the cervix and the uterine corpus reported in the literature, including the present case

First author	Year	Cases reported	Tumor type^a^
McGee	1962	1	Adenocarcinoma
Zaczek	1963	1	Adenocarcinoma
Buntine	1979	1	Adenocarcinoma
Valente & Susin	1987	1	Adenocarcinoma
Lang	1990	2	Adenocarcinoma
Ferry & Scully	1990	1	Adenocarcinoma
Stewart	1993	1	Adenocarcinoma
Yamamoto	1995	1	MMMT
Clement	1995	7	4/7 adenocarcinomas 3/7 MMMT
Silver	2001	11	9/11 adenocarcinomas 2/11 MMMT
Ordi	2001	1	Adenocarcinoma (uterine corpus)
Angeles	2004	1	Adenocarcinoma
Bagué	2004	6	3/6 adenocarcinomas 3/6 MMMT
Marquette	2006	1	Adenocarcinoma (uterine corpus)
Yap	2006	1	Adenocarcinoma
Fukunaga	2008	1	Adenocarcinoma
Wani	2008	1	Adenocarcinoma (uterine corpus)
Anagnostopoulos	2012	1	Adenocarcinoma
Nomoto	2012	2	Adenocarcinoma
Kenny	2012	8	Adenocarcinoma; 7 in the cervix and 1 in the corpus
Meguro	2013	1	MMMT
Menon	2013	1	Adenocarcinoma
Abdul-Ghafar	2013	1	Adenocarcinoma
Wu	2014	2	Adenocarcinoma (both in the uterine corpus)
Roma	2014	1	MMMT
Tseng	2014	1	MMMT
Mirkovic	2015	3	Adenocarcinoma
Tekin	2015	1	Adenocarcinoma
Zhao	2015	2	Adenocarcinoma (both in the uterine corpus)
Dierickx	2016	1	Adenocarcinoma
Yeo	2016	1	Adenocarcinoma
Ditto	2016	1	Adenocarcinoma
Puljiz	2016	1	Adenocarcinoma
Kim	2016	1	Adenocarcinoma (inthe uterine corpus)
Kir	2016	1	Adenocarcinoma
Ando	2017	1	Adenocarcinoma (confined to the myometrium)
Cavalcanti	2017	1	Mixed adenocarcinoma and high-grade neuroendocrine carcinoma
Ribeiro	2019	1	MMMT
Present case	2019	1	Adenocarcinoma

^a^ If not otherwise specified, the tumors arose in the cervix. MMMT: malignant mixed mesonephric tumor

## Case presentation

**Fig. 1:**
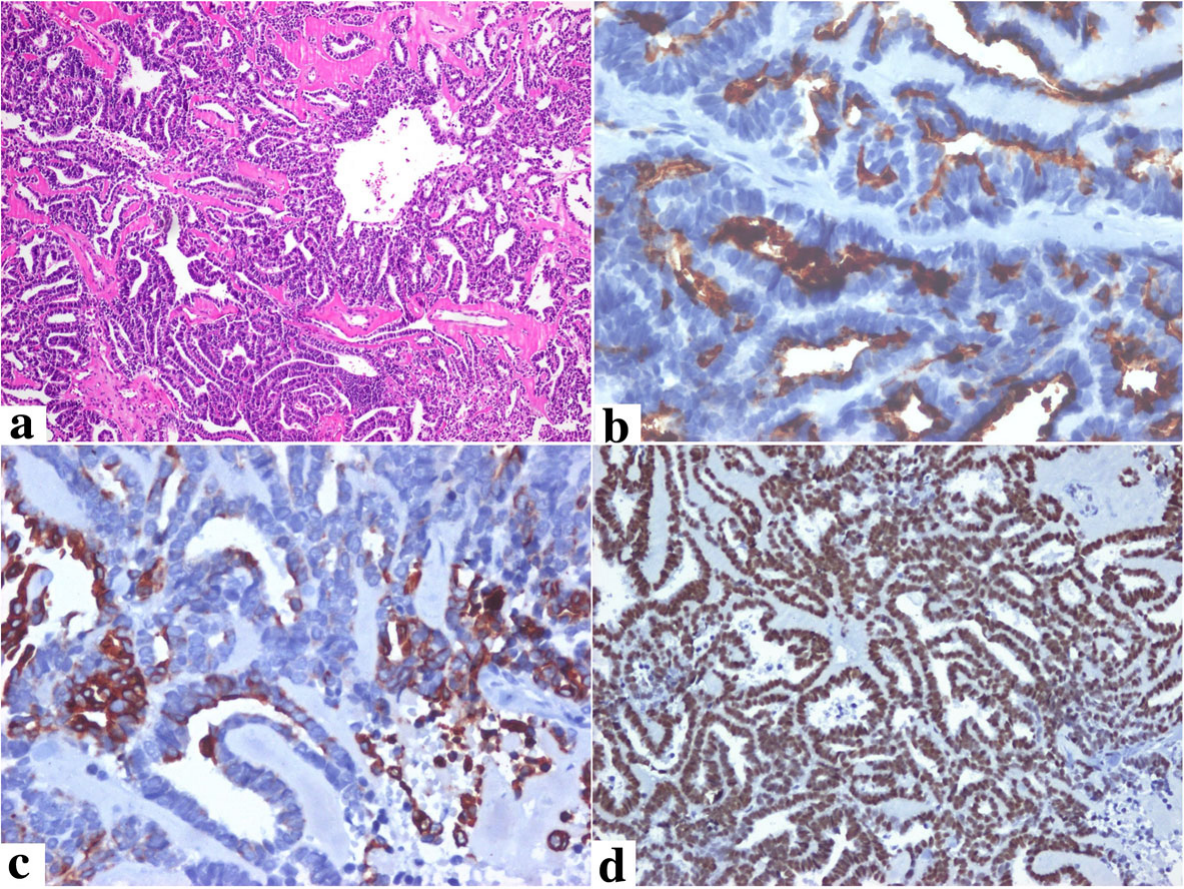
Mesonephric Adenocarcinoma of the Cervix. Epithelial neoplasm with a tubular, ductal, and papillary growth pattern producing intraluminal eosinophilic secretory material, located on a densely hyalinized stroma [HE 20 X] (**a**). The tumor cells were positive for CD10 (luminal pattern), keratin 7, and PAX2 (**b**, **c**, **d**)

**Fig. 2:**
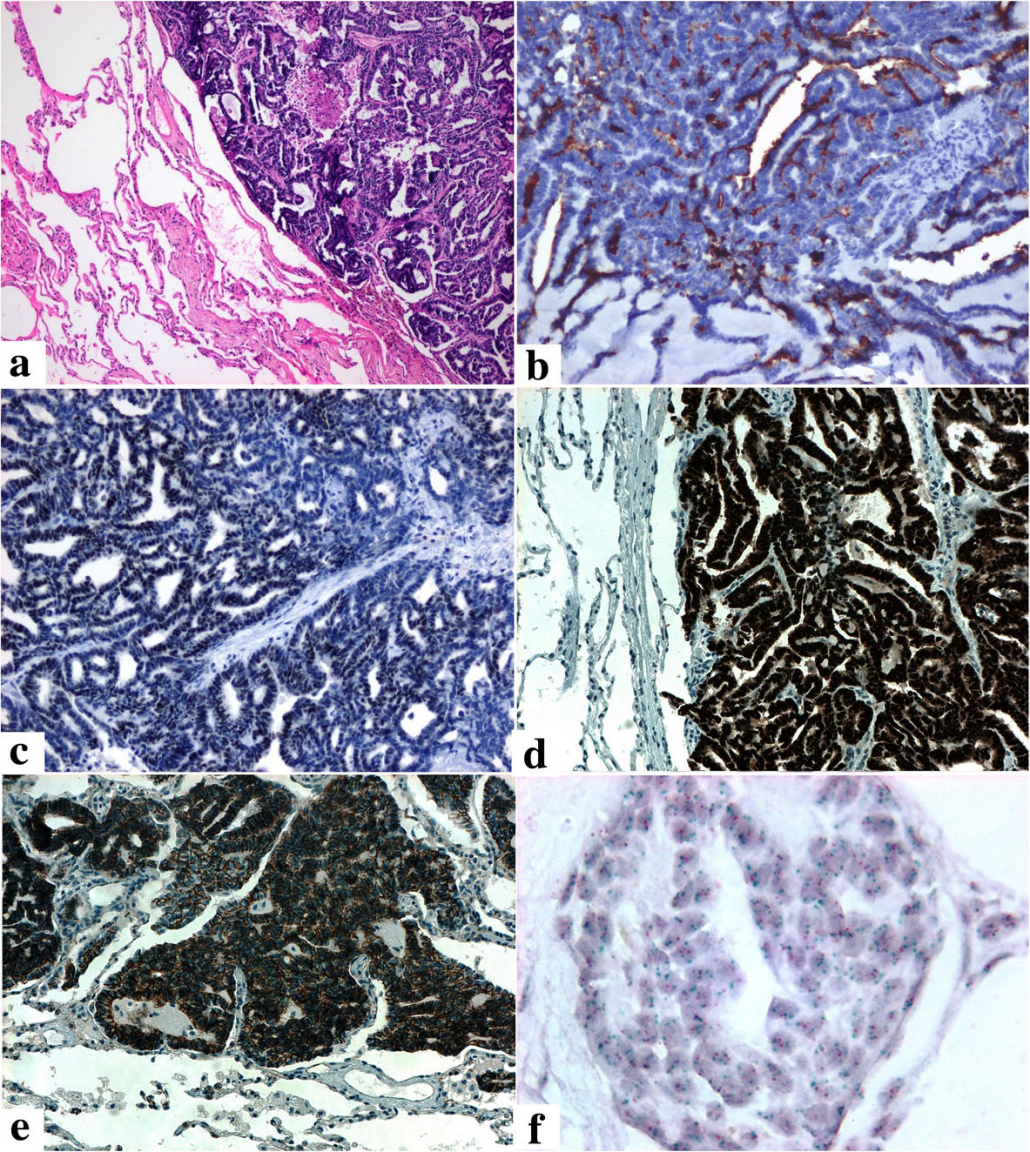
Lung metastasis of the endocervical mesonephric adenocarcinoma. Malignant tumor with a tubular, ductal, and papillary histological pattern (were very similar to the endocervical tumor) [HE 10X] (**a**). The neoplastic cells were positive for CD10 (luminal pattern), TTF-1, PAX-8 and Beta-catenin (membrane stain) (**b**, **c**, **d**, **e**). Chomogenic in Situ Hybridization (CISH) of 1q and 1p. Gain of 1q (green) and normal copy number of 1p (red) are shown, with one to two copies of 1p36 and three to eight copies of 1q25 (**f**)

An asymptomatic 48-year-old Hispanic female patient presented with a polypoid mass protruding into the endocervical canal during a gynecological examination in April 2014. The lesion had a cerebroid appearance during biopsy. Microscopic study revealed an epithelial neoplasm with a tubular, ductal, and papillary growth pattern producing intraluminal eosinophilic secretory material, located on a densely hyalinized stroma (Fig. 1a). The tumor cells were positive for CD10 (luminal pattern), p16INK4a (non-block staining pattern), PAX2 (Fig. 1b, c, d), inhibin, cytokeratin 7, WT-1, wild-type p53 (images not shown), and negative for estrogen receptors, progesterone receptors, cytokeratin 20, CEAm, and calretinin (images not shown). The Ki-67 index of the tumor was around 46%. This histological and immunophenotypic picture confirmed the diagnosis of mesonephric adenocarcinoma of the endocervix. With this diagnosis, the patient underwent a total hysterectomy outside the institution. Three years later, the patient presented pulmonary nodules in the lingula and left basal lobe that were resected by thoracotomy. The histological pattern (tubular, ductal, and papillary) (Fig. 2 a) and the immunohistochemical profile (CD10, TTF-1, PAX8, Beta-catenin (membrane pattern) (Fig. 2 b, c, d, e), PAX2 and p16 positive) of the pulmonary nodules correlated to those of the endocervical tumor. PAX8 staining was performed in order to document the gynaecological origin of the lung nodules [5, 6]. These findings confirmed metastasis of the endocervical mesonephric adenocarcinoma. The tumor was subjected to a multiple gene study using next-generation sequencing (NGS) technology (FoundationOneTM) to find therapeutic targets in our patient. Genomic alterations were identified in KRAS (G12C) and CTNNB1 (G34R). Additional findings were absence of microsatellite instability and a low tumor mutation burden with three mutations per megabase (TMB-Low, 3 Muts/Mb). Copy number analysis by CISH using the SPEC 1p36 and SPEC 1q25 Dual color probe (Zytovision) identified gain of chromosome 1q (Fig. 2f).

## Materials and methods

Immunohistochemical staining was performed on 4 μm formalin-fixed and paraffin-embedded (FFPE) tissue sections using VENTANA BenchMark system (Roche, Tucson, AZ) according to standard laboratory procedures. The following antibodies were used in the diagnostic work-up: Beta-catenin, anti-CD10, Calretinin, Cytokeratin 7, Cytokeratin 20, Estrogen receptor, Inhibin-alpha, p16INK4A, Progesterone receptor, PAX-2, PAX-8 and WT-1. Also we used CEAm, p53, and TTF-1 (BioGenex, Fremont, CA.)

### CISH analysis

Chromogenic in situ hybridization (CISH) analysis was performed using ZytoVision (Bremerhaven, Germany), Zyto*Dot*® 2C SPEC 1p36/1q25 dual color probe for assessing gain of chromosome 1q. CISH analysis was performed on 4 μm FFPE slides to detect cytogenetic aberrations associated with MNAC, following standard laboratory procedures. A total of 100 cells nuclei were counted by two pathologists independently.

### Molecular profiling

Comprehensive genomic profiling test with the FoundationOne panel of genes was performed by Foundation Medicine, Inc. (Cambridge, MA) based on published methods. FoundationOne is designed to include all genes known to be somatically altered in human solid tumors that are validated targets for therapy, either approved or in clinical trials, and /or that are unambiguous drivers of oncogenesis based on current knowledge. The current assay evaluates 315 genes, including introns of 28 genes involved in rearrangements.

## Discussion

Early in embryological development, two mesonephric (Wolffian) ducts begin to form and connect the mesonephros to the cloaca around the fourth week of gestation. In the presence of testosterone, these ducts will give rise to the epididymis, seminal vesicles, vas deferens, and ejaculatory ducts in males. In females, the mesonephric ducts regress. However, vestiges of these structures may persist along the female genital tract in the form of epithelial inclusions – the so-called mesonephric remnants – that may be found adjacent to the ovarian hilum, in the thickness of the broad ligament, in the vagina and, more frequently, in the lateral walls of the cervix [7]. The prevalence of mesonephric remnants varies from 1 to 22% in adults and up to 40% in children [8, 9].

The epithelia of the mesonephric remnants may expand into benign or malignant lesions (See Table 2). There is a biphasic variant of mesonephric adenocarcinoma with a sarcomatoid component that can display homologous or heterologous differentiation, named malignant mixed mesonephric tumor (MMMT) [9, 10]. Typically, a homologous component resembling either endometrial stroma or a non-specific spindle cell sarcoma is found in the setting of MMMT, although heterologous elements such as atypical cartilage, osteosarcoma, and rhabdomyosarcoma have been described [11, 12].

**Table 2:** Mesonephric-derived entities: Benign and malignant lesions

Entity	Clinical features	Pathological features				Main differential diagnoses
		Gross characteristics	Microscopic/morphological characteristics	IHC	Molecular features	
*Mesonephric remnants (MRs)*	Typically identified in asymptomatic women in reproductive and postmenopausal age groups. MRs can be seen in up to 22% of adults and 40% of newborns and children. The lateral wall of the cervix (3 and 9 o’clock) is the most frequent location. Not associated with increased risk of malignancy.	MRs are non-mass forming and thus are not clinically or grossly apparent.	Clusters or linear arrays of small tubules lined by bland cuboidal epithelia, lacking mucin.	PAX8, GATA3, and CD10 (+); calretinin 10% (+); ER, PR, p16, and p53 (−)	No studies have evaluated molecular alterations.	Mesonephric hyperplasia, endometrial adenocarcinoma with cervical stroma invasion.
*Gartner’s duct cyst (mesonephric cyst)*	Uncommon (< 1%); typically located in the lateral or anterior wall of the vagina. May be associated with renal and ureteral abnormalities. No increased risk of malignancy.	Presentation is similar to other vaginal cysts.	Bland, cuboidal to low columnar non-mucinous epithelia	CD10, GATA3, PAX8, and calretinin (+)	No studies have evaluated molecular alterations.	Müllerian cysts, Bartholin duct cysts (showing mucinous epithelia)
*Mesonephric hyperplasia (MH)*	Usually an incidental microscopic finding in reproductive and postmenopausal age groups. May be rarely associated with erosion, nodularity, or an abnormal Pap smear.	Usually not apparent on gross examination. Occasional thickening of the cervical wall. Formation of a discrete mass is rare.	Similar to mesonephric remnants, the proliferations are larger (> 6 mm) and more numerous, with more extensive involvement of the cervix. The most common type is a lobular variant.	PAX8, GATA3, and CD10 (+); calretinin 10% (+); ER, PR, p16, and p53 (−)	Activating *KRAS* and *NRAS* mutations are not found.	Mesonephric adenocarcinoma, endometrial adenocarcinoma with invasion of the cervical stroma, endocervical adenocarcinoma
*Mesonephric adenocarcinoma*	The vast majority of cases arise in the uterine cervix. Represents less than 1% of all carcinomas at this site. Patients commonly present with abnormal bleeding and/or an exophytic polypoid mass protruding into the cervical canal.	Firm mass in the lateral wall of the cervix. Diffusely thickened cervix may be an alternative presentation.	Often widely infiltrative. May display a variety of patterns: ductal, tubular, solid, papillary, retiform, and sex cord–like. Depending on the pattern, epithelial cells may be cuboidal or columnar. Rare cases are biphasic tumors, which disclose a sarcomatoid component.	CD10, CK7, PAX2, and PAX8 (+); GATA3 (+), but to a lesser extent compared with GATA 3 results in MRs and MH; TTF-1, calretinin, and inhibin are variably (+); CEAm, ER, and PR (−)	Canonical activating *KRAS* mutations, *NRAS* mutations, gain of 1q, no microsatellite instability. *TP53* mutations are variably present.	Mesonephric hyperplasia, endometrioid adenocarcinoma, mesonephric-like adenocarcinoma, clear-cell carcinoma, serous carcinoma

Mesonephric adenocarcinoma is a rare, non-mucinous cervical tumor. It accounts for less than 1% of all tumors at this site and is not related to human papillomavirus (HPV). It is usually located deep in the lateral cervical stroma, but rare cases of primary vaginal and uterine corpus MNAC have been reported [13, 14]. The average age of presentation is 53 years, and there is no apparent peak, since its prevalence in age groups from the third through the sixth decade is similar, with 26% of patients being younger than 40 years. Diagnosis is usually made on biopsy specimens, endometrial curettings, or hysterectomy specimens. Clinically, this type of tumor usually presents as abnormal vaginal bleeding or as a cervical mass on pelvic examination. Tumors may be discovered incidentally in some cases; less commonly, they may involve the entire cervical circumference, presenting as a barrel-shaped cervix. On gross examination, mesonephric adenocarcinoma may be in the form of an exophytic, nodular, or friable polypoid mass [3, 15, 16]. In our case, the patient presented with a polypoid mass protruding into the cervical canal with no abnormal bleeding.

Histologically, MNAC is usually widely infiltrative and may display numerous architectural growth patterns (See Table 2). The tubular pattern consists of back-to-back, small, round to oval glands that are closely packed together and lined by low columnar, cuboidal, or flattened cells, with some lumens containing dense eosinophilic secretions such as those seen in mesonephric remnants [17, 18]. In the ductal pattern, the tumor exhibits large glandular spaces with occasional intraluminal infoldings or papillae, lined by one to several layers of tall columnar cells with hyperchromatic nuclei. The retiform pattern is characterized by elongated, slit-like branching tubules variably containing intraluminal papillae with hyalinized fibrous cores and zigzag shaped glandular spaces resembling the rete ovarii [3, 19, 20]. A variation of the retiform pattern is a sieve-like pattern with cystic spaces lined by flattened cells. Cysts may be empty or contain colloid-like material. The sex cord–like pattern consists of cells growing in cords and trabeculae with scant cytoplasm [18]. A pathological parameter regarding solid/spindled morphology portends a worse prognosis.

Cytologically, the tumors disclose relatively uniform columnar cells with scant eosinophilic cytoplasm. The nuclei are usually oval and often have irregular membranes and frequent grooving. Nuclear pseudoinclusions may be also found, as well as mild to moderate atypia [7]. Marked nuclear atypia is not seen. Prominent nucleoli may occasionally be present. The mitotic index ranges from 1 to 50 mitoses per 10 HPFs (high-power fields) and may vary from case to case [3, 21]. Mesonephric hyperplasia of the lobular or diffuse pattern may be present in the background of MNAC.

Immunohistochemically, mesonephric adenocarcinoma is usually diffusely and strongly positive for CD10 (apical and luminal), CK7, PAX8, EMA (epithelial membrane antigen), and vimentin. PAX2 is usually positive, but a strong and diffuse expression is more likely to be associated with benign mesonephric lesions [22]. Other markers including calretinin, inhibin, and androgen receptors (AR) are variably positive. CEAm and CK20 are consistently negative markers. ER and PR are uniformly negative or only focally positive in MNAC [23–25]. No immunoprofile is diagnostic, but positive immunostaining for CD10, CK7, and calretinin along with negative immunostaining for CEAm is suggestive of mesonephric adenocarcinoma [20]. Although TTF-1 (thyroid transcription factor 1) is generally considered a biomarker for lung and thyroid carcinoma, it may be positive in MNAC. In our case, immunostaining of the metastatic specimen from the lingula showed positivity for TTF-1 and PAX8, the latter confirming the gynaecological origin of the metastasis (See Table 3). HNF1B (hepatocyte nuclear factor 1-beta), while considered a marker of clear cell carcinoma, may be expressed in a subset of mesonephric adenocarcinoma [26]. MNAC usually shows negative or weak focal staining for p16, which does not correlate with the presence of HPV. This p16 staining pattern correlates with the one seen in our case.

**Table 3:** Immunohistochemical and in situ hybridization for both the primary and the metastatic tumors

	Endocervical Tumor	Lung Tumor
Beta-catenin	NP	+ (membrane stain)
Calretinin	–	–
CEAm	–	NP
CD10	+ (luminal pattern)	+ (luminal pattern)
Cytokeratin AE1/AE3	NP	+
Cytokeratin 7	+	NP
Cytokeratin 20	–	NP
Estrogen Receptor	–	–
Inhibin, alpha	+	–
p16INK4A	+ (Non-block)	+ (Non-block)
p53	Negative (Wild type)	NP
PAX-2	+	+
PAX-8	NP	+
Progesterone Receptor	–	NP
TTF-1	NP	+
Vimentin	NP	+
WT-1	+	+
1q	NA^a^	Gain

^a^ CISH was performed on the available tissue sample form the endocervical tumor. However, the results were not satisfactory and no analysis could be carried out

*KRAS*/*NRAS* mutations are the most common molecular alterations detected in mesonephric adenocarcinomas. MNAC is characterized by recurrent *KRAS* mutations [22]. *KRAS* mutations have also been documented in mesonephric-like adenocarcinomas of the female genital tract [16]. Besides, *KRAS* are more common than *NRAS* mutations*,* and the two are mutually exclusive. The chromatin remodeling genes *ARID1A/B* are frequently mutated as well. Common genetic aberrations found in endometrial and other types of cervical adenocarcinoma, such as *PTEN* and *PIK3CA*, are not reported in MNAC. *TP53* is uncommonly mutated in mesonephric adenocarcinoma and other cervical adenocarcinomas, while more than 90% of endometrial serous carcinomas harbor *TP53* aberrations. Thus, *KRAS* or *NRAS* mutation in combination with the lack of *PIK3CA*, *PTEN*, and *TP53* mutations would support a diagnosis of mesonephric adenocarcinoma [27, 28].

Different copy number variations (CNVs) have been reported in MNAC. A gain of 1q is the most common CNV associated with MNAC. In the series reported by Mirkovic et al. [22] and Na et al. [18], a 1q gain was detected in 12 out of 17 cases and 11 out of 12 cases, respectively. Interestingly, a 1q gain is also the most common copy number alteration among endometrial carcinomas [29]. We identified a gain of 1q by CISH and calculated the ratio of hybridization signals for 1p36 and 1q25 on 100 tumoral nuclei (3.8 for 1q25 and 1.8 for 1p36). It is worthy of note that focal amplification of 1q may influence the oncogenic potential of tumor cells. Approximately 10% of all cancers show a focal amplification of chromosome 1q21.2, a region harboring the antiapoptotic gene MCL1 [30]. In addition, in cases of multiple myeloma, many other genes located on the proximal region of chromosome 1q, such as *CKSB1* and *PDZ1*, have proven to portend a worse prognosis and resistance to certain chemotherapy agents. Regarding MNAC, current data suggest that a 1q as well as a 10q gain may be indicators of aggressive behavior and may increase the risk of developing metastasis [18, 22]. Other known arm-level chromosomal abnormalities included loss of chromosomes 1p and 9p and gain of chromosomes 10 and 19 [18, 22]. Notably, no evidence of microsatellite instability or hypermutation has been identified in MNAC.

To the best of our knowledge, alterations of *CTNNB1* in mesonephric adenocarcinoma have not been documented so far. In our case, next-generation sequencing technology detected *KRAS* G12C and *CTNNB1* G34R mutations, absence of microsatellite instability and low tumor mutation burden (three mutations per megabase). This is the first case of MNAC reporting *CTNNB1* gene mutation.

The *CTNNB1* gene is located on the short arm of chromosome 3 and encodes beta-catenin 1. This protein is part of a molecular complex related to the adherens junctions of epithelial cells and maintenance of cell adhesion [31, 32]. Mutation and deletion of this gen were each reported in only 2% of cases of cervical squamous cell carcinoma and endocervical adenocarcinoma in the TCGA (The Cancer Genome Atlas) database.

Beta-catenin 1 is part of the Wnt cell signaling pathway. Specifically, Wnt4 is involved in inhibiting the differentiation of mesonephric duct–derived tissue, during gonadal development [33]. Normally, activation of the Wnt pathway induces cytoplasmic accumulation of free beta-catenin 1 and the consequent expression of target genes. When not activated, the *CK1* (casein kinase 1) and *GSK3*β (glycogen synthase kinase 3β) kinases phosphorylate specific amino acids in beta-catenin 1 and signal it for degradation in the proteasome. These amino acids are encoded by a region of the *CTNNB1* gene located in exon 3, which was indeed mutated in our case [34, 35]. This mutation leads to a constitutive stabilization of beta-catenin 1, inducing cell proliferation and decreasing intercellular adhesion. Therefore, we speculate that the mutation found in this case could be related to the development of mesonephric adenocarcinoma because of a failure in the inhibition of mesonephric duct–derived tissue. Moreover, we think it is possible that there is a relationship between the mutation described and the presence of pulmonary metastasis in this case. It is interesting that a case of malignant mesonephric tumor with pulmonary metastasis as initial clinical presentation has been reported, but no molecular alterations were sought or informed [36].

However, there is no saying whether the mutation of *CTNNB1* is an early phenomenon in tumorigenesis contributing to the appearance and growth of the tumor or a later event caused by the accelerated cell division characteristic of neoplasms. Furthermore, it would be necessary to analyze the role of the *CTNNB1* mutation in the set of molecular alterations presented in this case (*KRAS* gene mutation and 1q gain). To determine whether there is a relationship between oncogenesis and the development of metastasis with the mutation described, it would be necessary to expand the study of *CTNNB1* gene mutation (especially activating mutations that affect exon 3) in other reported cases of mesonephric adenocarcinoma.

In this context, it is interesting to note that a FATWO (female adnexal tumor of probable Wolffian origin) case has been reported in which a missense mutation was identified in the *CTNNB1* gene. However, of the three tumors included in the study, it was the only one that showed this gene mutation [37].

On the other hand, mutations of the *CTNNB1* have been associated with other malignant tumors, such as melanoma, renal cell carcinoma, hepatocarcinoma, medulloblastoma, colon cancer, lung cancer, and ovarian cancer, among others [31, 38, 39].

MNAC has a broad differential diagnosis. On the benign spectrum, it should be differentiated from MH and MR (See Table 2). Ki-67 immunostaining may be helpful, since it has been reported to show positivity in only 1–2% of cells in MH versus 5–20% or more in carcinoma [40, 41]. In our case, the percentage of Ki-67 positive cells in the primary tumor was 46%. On the malignant spectrum, the ductal variant of the tumor should be differentiated from endometrioid adenocarcinoma, which is usually positive for ER, PR, and vimentin. Tumors with papillary and slit-like arrangements can be confused with serous carcinoma, which is CEAm (+), ER (−), and PR (−). Primary cases are very rare, and most often represent metastasis from ovarian serous carcinoma. Foci of hobnail cells in MNAC can resemble clear-cell carcinoma, which is characteristically Bcl-2 (+). Medium-size or duct-like formations may also mimic endometrioid adenocarcinoma [28]. In contrast to MNAC, these HPV-negative cervical tumors show high nuclear grade.

Mesonephric adenocarcinomas can be very aggressive, even when low stage. Yap et al. reviewed a total of 31 cases of MNAC in which most patients (82%) presented at International Federation of Gynecology and Obstetrics (FIGO) stage 1B; one third of FIGO stage 1 patients developed recurrence even after curative resection; and one fifth of the patients with stage 1B disease had a fatal course between 1 and 9 years after diagnosis [4]. Local recurrence and distant metastases were common findings in this study, and median and mean times to recurrence were 2.1 and 3.6 years, respectively. In another series, Dierickx et al. reported a recurrence rate of 32% in patients with stage 1 MNAC [1]. Most patients died within one year after recurrence, despite therapy. Nevertheless, MNAC may have a better prognosis than Müllerian counterparts [2]. For most patients, treatment consists of hysterectomy, bilateral salpingo-oophorectomy, and pelvic lymphadenectomy, depending on the stage of disease at diagnosis.

A malignant clinical course has been reported in about 40% of MNAC cases. Distant metastases at initial diagnosis are detected in less than 5% of patients [18]. In our case, the patient had no metastasis at the time of diagnosis. Mirkovic et al. reported that all patients with MNAC and confirmed metastasis (four out of sixteen patients) had a *KRAS* mutation, as in our case. *TP53* mutations were not present in any of the tumors of metastatic patients. Notably, most of the metastatic tumors (75%) exhibited gains of chromosomes 10 and 12, and none of the non-metastatic cases had this finding. Metastases were more common when the tumor exhibited a sarcomatous component [4]. Frequent sites of distant metastases included bones, lungs, pleura, and liver. In our case, metastasis to the left lung developed three years after diagnosis in absence of a spindle-cell component in the tumor.

## Conclusion

To the best of our knowledge, this is the first case of MNAC of the cervix reporting a mutation in *CTNNB1* gene. Further analysis is necessary to determine whether this mutation has a role in oncogenesis or in metastases development in the setting of this neoplasia.

## Data Availability

Not applicable.

## Abbreviations

AR: Androgen receptors
CEAm: Carcinoembryonic antigen monoclonal
CISH: Chromogenic in situ hybridization
CK1: Casein kinase 1
EMA: Epithelial membrane antigen
ER: Estrogen receptors
FATWO: Female adnexal tumor of probable Wolffian origin
GSK3β: Glycogen synthase kinase 3β
HNF1B: Hepatocyte nuclear factor 1-beta
HPF: High-power field
HPV: Human papillomavirus
MH: Mesonephric hyperplasia
MMMT: Malignant mixed mesonephric tumor
MNAC: Mesoneprhic adenocarcinoma
MRs: Mesonephric remnants
Muts/Mb: Mutations per megabase
NGS: Next-generation sequencing
PR: Progesterone receptors
TCGA: The cancer genoma atlas
TMB: Tumor mutation burden
TTF-1: Thyroid transcription factor 1

## References

[1] Dierickx A, Göker M, Braems G, Tummers P, Van den Broecke R (2016). Mesonephric adenocarcinoma of the cervix: case report and literature review. Gynecol Oncol Rep.

[2] Tekin L, Yazici A, Akbaba E, Akin MN (2015). Mesonephric adenocarcinoma of the uterine cervix: a case report and review of the literature. J Pak Med Assoc.

[3] Pirog EC (2010). Diverse facets of cervical adenocarcinoma: comprehensive review of clinicopathologic features and diagnostic criteria. Diagnostic Histopathol.

[4] Yap OWS, Hendrickson MR, Teng NNH, Kapp DS (2006). Mesonephric adenocarcinoma of the cervix: a case report and review of the literature. Gynecol Oncol.

[5] Yemelyanova A, Gown AM, Wu L-S-F, Holmes BJ, Ronnett BM, Vang R (2014). PAX8 expression in uterine adenocarcinomas and mesonephric proliferations. Int J Gynecol Pathol.

[6] Wong S, Hong W, Hui P, Buza N (2017). Comprehensive analysis of PAX8 expression in epithelial malignancies of the uterine cervix. Int J Gynecol Pathol.

[7] Howitt BE, Nucci MR (2018). Mesonephric proliferations of the female genital tract. Pathology..

[8] Cavalcanti MS, Schultheis AM, Ho C (2017). Mixed mesonephric adenocarcinoma and high-grade neuroendocrine carcinoma of the uterine cervix: case description of a previously unreported entity with insights into its molecular pathogenesis. Int J Gynecol Pathol.

[9] Ferry JA, Scully RE (1990). Mesonephric remnants, hyperplasia, and neoplasia in the uterine cervix. A study of 49 cases. Am J Surg Pathol.

[10] Seidman JD, Tavassoli FA (1995). Mesonephric hyperplasia of the uterine cervix: a clinicopathologic study of 51 cases. Int J Gynecol Pathol.

[11] Young RH, Clement PB (2002). Endocervical adenocarcinoma and its variants: their morphology and differential diagnosis. Histopathology..

[12] Bagué S, Rodríguez IM, Prat J (2004). Malignant mesonephric tumors of the female genital tract: a clinicopathologic study of 9 cases. Am J Surg Pathol.

[13] Roma AA (2014). Mesonephric carcinosarcoma involving uterine cervix and vagina: report of 2 cases with immunohistochemical positivity for PAX2, PAX8, and GATA-3. Int J Gynecol Pathol.

[14] Wani Y, Notohara K, Tsukayama C (2008). Mesonephric adenocarcinoma of the uterine corpus: a case report and review of the literature. Int J Gynecol Pathol.

[15] Howitt BE, Emori MM, Drapkin R (2015). GATA3 is a sensitive and specific marker of benign and malignant mesonephric lesions in the lower female genital tract. Am J Surg Pathol.

[16] Mirkovic J, McFarland M, Garcia E (2018). Targeted genomic profiling reveals recurrent KRAS mutations in mesonephric-like adenocarcinomas of the female genital tract. Am J Surg Pathol.

[17] Fawcett KJ, Dockerty MB, Hunt AB (1966). Mesonephric carcinoma of the cervix uteri: a clinical and pathologic study. Am J Obstet Gynecol.

[18] Na K, Kim H-S (2019). Clinicopathologic and molecular characteristics of mesonephric adenocarcinoma arising from the uterine body. Am J Surg Pathol.

[19] Baghai F, Yazdani F, Etebarian A, Garajei A, Skalova A (2017). Clinicopathologic and molecular characterization of mammary analogue secretory carcinoma of salivary gland origin. Pathol Res Pract.

[20] Ordi J, Romagosa C, Tavassoli FA (2003). CD10 expression in epithelial tissues and tumors of the gynecologic tract: a useful marker in the diagnosis of mesonephric, trophoblastic, and clear cell tumors. Am J Surg Pathol.

[21] Gupta OP, Guirguis MN, Diejomaoh F (1988). Mesonephric adenocarcinoma of cervix: a case report. Int J Gynaecol Obstet.

[22] Mirkovic J, Sholl LM, Garcia E (2015). Targeted genomic profiling reveals recurrent KRAS mutations and gain of chromosome 1q in mesonephric carcinomas of the female genital tract. Mod Pathol.

[23] Heatley MK (2007). Immunohistochemical and functional biomarkers of value in female genital tract lesions: a systematic review with statistical meta-analysis. Int J Gynecol Pathol.

[24] McCluggage WG (2006). Immunohistochemical and functional biomarkers of value in female genital tract lesions. Int J Gynecol Pathol.

[25] Nofech-Mozes S, Khalifa MA, Ismiil N (2008). Immunophenotyping of serous carcinoma of the female genital tract. Mod Pathol.

[26] Kenny SL, McBride HA, Jamison J, McCluggage WG (2012). Mesonephric adenocarcinomas of the uterine cervix and corpus: HPV-negative neoplasms that are commonly PAX8, CA125, and HMGA2 positive and that may be immunoreactive with TTF1 and hepatocyte nuclear factor 1-β. Am J Surg Pathol.

[27] Goyal A, Yang B (2014). Differential patterns of PAX8, p16, and ER immunostains in mesonephric lesions and adenocarcinomas of the cervix. Int J Gynecol Pathol.

[28] Kır G, Seneldir H, Kıran G (2016). A case of mesonephric adenocarcinoma of the uterine cervix mimicking an endometrial clear cell carcinoma in the curettage specimen. J Obstet Gynaecol.

[29] Micci F, Teixeira MR, Haugom L, Kristensen G, Abeler VH, Heim S (2004). Genomic aberrations in carcinomas of the uterine corpus. Genes Chromosom Cancer.

[30] Beroukhim R, Mermel CH, Porter D (2010). The landscape of somatic copy-number alteration across human cancers. Nature..

[31] Stewart CA, Wang Y, Bonilla-Claudio M (2013). CTNNB1 in mesenchyme regulates epithelial cell differentiation during Müllerian duct and postnatal uterine development. Mol Endocrinol.

[32] Betancur P, Bronner-Fraser M, Sauka-Spengler T (2010). Assembling neural crest regulatory circuits into a gene regulatory network. Annu Rev Cell Dev Biol.

[33] Pellegrino M, Maiorino R, Schonauer S (2010). WNT4 signaling in female gonadal development. Endocr Metab Immune Disord Drug Targets.

[34] Cho K-H, Baek S, Sung M-H (2006). Wnt pathway mutations selected by optimal β-catenin signaling for tumorigenesis. FEBS Lett.

[35] Shang S, Hua F, Hu Z-W (2017). The regulation of b-catenin activity and function in cancer: therapeutic opportunities. Oncotarget..

[36] Yeo MK, Choi SY, Kim M, Kim KH, Suh KS (2016). Malignant mesonephric tumor of the cervix with an initial manifestation as pulmonary metastasis: case report and review of the literature. Eur J Gynaecol Oncol.

[37] Cossu A, Casula M, Paliogiannis P (2017). Female adnexal tumors of probable Wolffian origin (FATWO): a case series with next-generation sequencing mutation analysis. Int J Gynecol Pathol.

[38] Yang C-M, Ji S, Li Y, Fu L-Y, Jiang T, Meng F-D (2017). β-Catenin promotes cell proliferation, migration, and invasion but induces apoptosis in renal cell carcinoma. Onco Targets Ther.

[39] Bi R, Bai Q-M, Yang F (2015). Microcystic stromal tumour of the ovary: frequent mutations of β-catenin (CTNNB1) in six cases. Histopathology..

[40] Fukunaga M, Takahashi H, Yasuda M (2008). Mesonephric adenocarcinoma of the uterine cervix: a case report with immunohistochemical and ultrastructural studies. Pathol Res Pract.

[41] Silver SA, Devouassoux-Shisheboran M, Mezzetti TP, Tavassoli FA (2001). Mesonephric adenocarcinomas of the uterine cervix: a study of 11 cases with immunohistochemical findings. Am J Surg Pathol.

